# Anticoagulation Management: Current Landscape and Future Trends

**DOI:** 10.3390/jcm14051647

**Published:** 2025-02-28

**Authors:** Andaleb Kholmukhamedov, David Subbotin, Anna Gorin, Ruslan Ilyassov

**Affiliations:** 1 Versiti Blood Research Institute, Milwaukee, WI 53226, USA; 2 School of Dentistry, University of California Los Angeles, Los Angeles, CA 90095, USA; 3 School of Public Health, San Diego State University, San Diego, CA 92115, USA; 4 Scripps Memorial Hospital, Encinitas, CA 92024, USA

**Keywords:** coagulation, haemostasis, anticoagulants, thrombosis, heparin, warfarin, DOAC, FXIa inhibitor

## Abstract

Blood transports nutrients and oxygen to the cells while removing the waste. It also possesses a hemostasis function to prevent excessive bleeding. However, abnormal clot formation (thrombosis) within healthy blood vessels can lead to life-threatening conditions like heart attacks, strokes, and pulmonary embolism. This review explores anticoagulants, their historical aspects, current clinical applications, and future trends. Anticoagulants play a critical role in preventing and treating thrombosis by interfering with different stages of blood clotting. The journey began with heparin, a rapidly acting injectable medication discovered in 1916. The introduction of warfarin in the 1950s revolutionized anticoagulation by offering long-term oral regimens. Today, anticoagulants are crucial for managing conditions like deep vein thrombosis and pulmonary embolism, especially in an aging population with a rising prevalence of thrombotic complications. Three main types of anticoagulants are used today: vitamin K antagonists (VKAs), injectable heparins, and direct oral anticoagulants (DOACs). Despite advancements, managing anticoagulant therapy remains complex due to individual patient variability, the need for regular monitoring, and the delicate balance between preventing thrombosis and bleeding risks. Emerging trends include the development of factor XIa inhibitors, which promise more targeted thrombosis prevention with potentially lower bleeding risks. This review highlights the ongoing innovation in anticoagulant development, the need for precise management, and potential future avenues like factor XIa inhibitors. Additionally, artificial intelligence holds promise for improving patient outcomes and addressing the complexities of thrombotic disease management by personalizing therapy and reducing bleeding risks.

## 1. Introduction

Blood performs a remarkable feat. It continuously navigates a delicate balance: flowing freely to deliver oxygen and nutrients throughout the body while also possessing the ability to form clots and prevent life-threatening blood loss at injury sites. However, the formation of clots within healthy blood vessels, known as thrombosis, poses a significant threat. These clots can act like dams, obstructing blood flow and starving tissues of oxygen and nutrients. Depending on the location of the clot, this can have devastating consequences, leading to events like heart attacks (clots within cardiac vasculature), strokes (cerebral vasculature), pulmonary embolism (pulmonary vascular bed), etc.

Three major classes of pharmaceutical agents used to prevent and/or treat thrombosis are antiplatelets (primarily affecting primary hemostasis), anticoagulants, and fibrinolytic agents. They work through different mechanisms and target different components of the hemostatic process. This review focuses on anticoagulants, a class of medications affecting coagulation (secondary hemostasis) used to prevent and treat thrombosis. It will dive into their history, current use, and future directions, exploring how they help maintain this delicate balance within the body. Antiplatelets [1,2] and thrombolytic/fibrinolytic agents [3,4,5] are discussed elsewhere.

Aptly named, anticoagulants are medications that combat the formation of harmful blood clots (thrombi). They achieve this by targeting various steps in the clotting cascade. The development of anticoagulants dates back to the early 20th century, with the discovery of heparin in 1916 marking a significant milestone. Heparin, a naturally occurring anticoagulant, was first isolated from canine liver cells and later found widespread use due to its rapid onset and ability to be administered parenterally. The introduction of warfarin in the 1950s revolutionized oral anticoagulant therapy, offering an effective means of long-term anticoagulation. Originally developed as a rodenticide, warfarin’s potent anticoagulant properties were quickly adapted for clinical use.

Anticoagulation therapy requires careful selection and monitoring to maintain a delicate balance within the hemostatic system. Effective anticoagulation ensures smooth blood flow, prevents unwanted clots, and safeguards patients from life-threatening bleeding complications.

Several factors underline the growing importance of effective management:

- Increased Need: The rising prevalence of thrombotic diseases like deep vein thrombosis and pulmonary embolism necessitates wider use of effective anticoagulants.
- Aging Population: As populations age, the risk of blood clots naturally increases, making targeted anticoagulation crucial for this demographic.
- Advancements in Therapy: Improvements in medications and monitoring technologies have enhanced safety, efficacy, and personalized care in anticoagulation.

These factors outline the importance of ongoing innovation and precise management in this critical area of medicine.

## 2. Current Landscape of Anticoagulation Management

**Figure 1:**
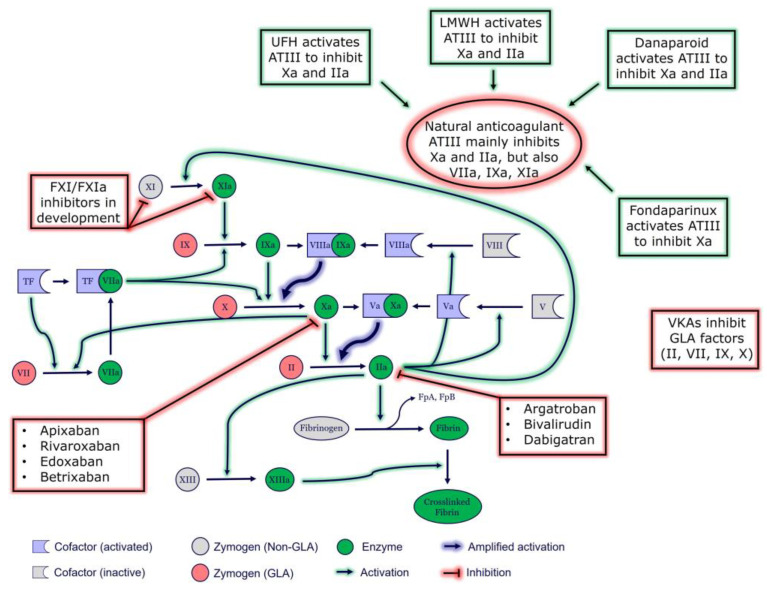
**Coagulation cascade and targets of anticoagulant medications**. ATIII, antithrombin III; FpA, fibrinopeptide A; FpB, fibrinopeptide B; GLA, Vitamin K-dependent carboxylation/gamma-carboxy glutamic domain; TF, tissue factor; VKA, vitamin K antagonist; UFH, unfractionated heparin.

There are several different pharmacological drugs in the world of anticoagulation, each with a unique mechanism of action and role in thrombosis prevention. This review explores several categories of anticoagulants currently used to treat and/or prevent thrombosis (see Table 1 and Figure 1). Regarding clinical anticoagulation, each category has unique pharmacokinetics, advantages, and disadvantages. Here, we will review the most used categories of anticoagulants.

### 2.1. Vitamin K Antagonists

Vitamin K antagonists (VKAs) mark the first class of oral anticoagulant therapies, with their origins tracing back to the pioneering work by Danish scientist Carl Peter Henrik Dam in 1929 [6]. In his investigation into chick nutrition, Dam noted a hemorrhagic syndrome in chicks fed a cholesterol-deficient diet, identifying the absent dietary factor as “Koagulationsvitamin”, later shortened to vitamin K. The narrative continued in the late 1930s in North America, where farmers observed severe bleeding in their cattle consuming spoiled sweet clover hay. Karl Paul Link at the University of Wisconsin in Madison pinpointed a toxic compound in the hay, a coumarin derivative, responsible for the bleeding [7,8]. This breakthrough set the stage for a new category of oral anticoagulants, the inception of the first vitamin K antagonist, and the subsequent global adoption of VKAs for the management of thrombotic complications.

VKAs exert their effect by inhibiting the hepatic gamma-carboxylation of vitamin K-dependent clotting factors (II, VII, IX, and X) through competition with vitamin K for vitamin K epoxide reductase complex 1 (VKORC1) binding sites. This results in the formation of so-called proteins induced by vitamin K absence (PIVKA). Factors without the gamma-carboxyl group have defective functions within the coagulation cascade, ultimately hindering thrombin generation and clot formation.

Among VKAs, warfarin is the most prevalent, offering a cost-effective anticoagulation strategy. However, its efficacy is challenged by individual response variability influenced by both genetic and environmental factors. Genetic considerations encompass polymorphisms in the VKORC1 gene and enzymes engaged in warfarin metabolism (e.g., CYP2C9), while environmental factors include dietary restrictions patients have to follow for the best VKA efficacy [9,10]. For VKA-treated patients, healthcare practitioners advocate for consistent monitoring and maintenance of a steady dietary intake of vitamin K to ensure predictable medication responses and mitigate the risks of under-anticoagulation (predisposing to increased clot risk) or over-anticoagulation (heightening bleeding risk). Nevertheless, patient adherence poses a significant challenge in clinical practice, necessitating regular monitoring of VKA effects using prothrombin time/international normalized ratio (PT/INR) to sustain therapeutic levels.

### 2.2. Injectable Anticoagulants—Heparins

Heparin, a naturally occurring sugar molecule (glycosaminoglycan), is a potent anticoagulant. Most heparins clinically used today come from animal sources like porcine intestinal mucosa or bovine lung tissue. Heparin was accidentally discovered in dog liver by McLean and Howell in 1916 and revolutionized the management of thrombotic disorders in the 20th century [11]. This substance, later named heparin after the Greek word “hepar” for liver, laid the foundation for what eventually became one of the most widely used anticoagulants [12].

Heparins act as a catalytic accelerator of antithrombin III (ATIII), a physiological inhibitor of thrombin and other coagulation factors, thus inhibiting the coagulation cascade. Upon administration or release by internal sources (primarily mast cells) [13], heparin binds to ATIII, inducing a conformational change that increases ATIII’s affinity to thrombin and factor Xa by approximately 1000-fold [14]. This augmentation of ATIII activity results in the inhibition of a series of coagulation cascade reactions, including the thrombin-mediated conversion of fibrinogen to fibrin, consequently impeding clot formation.

Heparin plays a pivotal role in the prevention and management of venous thromboembolism (VTE), including deep vein thrombosis (DVT) and pulmonary embolism (PE). Its rapid onset of action makes it indispensable in acute settings. Heparin is routinely used during cardiovascular surgeries, such as coronary artery bypass grafting (CABG) and percutaneous coronary intervention (PCI), to prevent intraoperative clot formation. In the setting of acute coronary syndromes (ACS), heparin is administered concomitantly with antiplatelet agents to prevent thrombus propagation and recurrent ischemic events [15,16]. Heparin is also employed as an anticoagulant during hemodialysis and extracorporeal membrane oxygenation (ECMO) to prevent clot formation within the extracorporeal circuits [17,18].

Heparins are predominantly administered parenterally via intravenous (IV) or subcutaneous (SC) routes. Intravenous administration is preferred in acute settings, whereas subcutaneous injections are employed for prophylactic anticoagulation. There are two major types of heparins: unfractionated heparin (UFH) and low molecular weight heparin (LMWH). UFH comprises heterogeneous chains of varying molecular weights. Its pharmacokinetic profile necessitates frequent monitoring by activated partial thromboplastin time (aPTT) or anti-Xa assay (more specific than aPTT) to maintain therapeutic anticoagulation. LMWH, derived from UFH through chemical or enzymatic depolymerization, exhibits more predictable pharmacokinetics and a lower risk of heparin-induced thrombocytopenia (HIT) [19,20]. Monitoring of LMWH is also performed by either aPTT or anti-Xa assays, but requirements are less stringent compared to UFH, and routine monitoring is typically not required, except in specific clinical scenarios.

The most common adverse effect of heparin therapy is bleeding, ranging from minor bruising to life-threatening hemorrhages. Close monitoring of hemostatic parameters is imperative to mitigate this risk of severe bleeding events. Another rare but potentially catastrophic complication of heparin use, heparin-induced thrombocytopenia (HIT), manifests as a paradoxical thrombotic state secondary to heparin-induced platelet activation. Prompt recognition and discontinuation of heparin are crucial in managing this condition.

Heparins remain to be an indispensable tool in the armamentarium of anticoagulant therapy. Its unparalleled efficacy in preventing and managing thrombotic disorders, coupled with meticulous monitoring and vigilant attention to potential adverse effects, underscore its enduring significance in contemporary medicine.

### 2.3. Direct Oral Anticoagulants

Unlike VKAs and heparins, the history of DOACs is relatively recent, with their emergence in the late 20th and early 21st centuries driven by advancements in pharmacological research, drug development, our understanding of the coagulation cascade, and the identification of specific pharmacological targets for anticoagulant intervention. The mechanism of action of DOACs differs from that of VKAs. DOACs directly inhibit key coagulation cascade components, specifically targeting either factor IIa (FIIa; thrombin) or factor Xa (FXa), pivotal enzymes involved in clot formation. By selectively inhibiting these enzymes, DOACs interfere with the conversion of fibrinogen to fibrin and the subsequent amplification of coagulation, thereby thwarting thrombus formation [21].

The advent of DOACs has introduced several agents into clinical practice, including dabigatran, rivaroxaban, apixaban, betrixaban, and edoxaban. Each DOAC exhibits unique pharmacokinetic and pharmacodynamic properties, influencing factors such as bioavailability, half-life, and pharmacoclearance. Consequently, the selection of a specific DOAC is guided by considerations such as the patient’s clinical profile, renal and/or hepatic function, and potential drug interactions.

Clinical trials have demonstrated the efficacy and safety of DOACs across various indications, including stroke prevention in atrial fibrillation, treatment of venous thromboembolism, and thromboprophylaxis in orthopedic surgery [22,23,24,25,26]. Compared to VKAs, DOACs offer several advantages, including rapid onset of action, predictable pharmacokinetics, and fewer dietary and drug interactions [27].

However, like all anticoagulants, DOACs are not without limitations. Adherence to prescribed dosing regimens is crucial to optimizing therapeutic outcomes and minimizing the risk of thrombotic or bleeding complications [28]. Additionally, monitoring the anticoagulant effect of DOACs differs from that of VKAs, with specialized laboratory assays such as anti-factor Xa activity or diluted thrombin time being utilized in specific clinical scenarios [29]. DOACs represent a significant advancement in anticoagulation therapy, offering clinicians and patients alternative treatment options with improved convenience and safety profiles compared to traditional VKAs. Ongoing research and clinical experience continue to refine our understanding of DOACs and their optimal utilization in various clinical settings.

### 2.4. Challenges in Current Anticoagulation Management

Balancing clot prevention with bleeding risk poses several challenges in current management strategies for thrombotic disorders and anticoagulant therapy. One of the key challenges is individual variability. Patients exhibit significant variability in their thrombotic and bleeding risks due to factors such as age, comorbidities, genetics, factor levels, concomitant medications, and lifestyle factors. Finding the optimal balance between preventing thrombosis and minimizing bleeding complications requires individualized risk assessment and tailored treatment approaches. For instance, patients with a history of gastrointestinal bleeding or certain genetic factors may have a heightened risk of bleeding, influencing the selection of a safer anticoagulant despite other clinical factors.

One of many challenges in anticoagulation management is gender-specific differences in anticoagulation, which are increasingly recognized as important factors influencing treatment outcomes. While the fundamental principles of anticoagulation remain the same, a number of sociobiological factors can contribute to variations in drug response, bleeding risk, and management strategies. For example, hormonal fluctuations associated with menstruation, pregnancy, and menopause can affect the pharmacokinetics and pharmacodynamics of anticoagulants, potentially altering their efficacy and safety profile. Studies have shown that women may experience a higher risk of certain bleeding complications, such as menorrhagia, while on anticoagulants [30,31,32]. Furthermore, differences in body weight can influence the dosing and monitoring of anticoagulants [33]. Recognizing and addressing these gender-specific differences is essential for improving the safety and effectiveness of anticoagulation therapy and ensuring equitable care for all patients.

Another challenge is the selection of the most appropriate anticoagulant for a given patient and clinical scenario. Traditional anticoagulants like warfarin and heparins have well-established efficacy but are associated with a narrow therapeutic window and require frequent monitoring. DOACs, such as dabigatran, offer advantages in terms of convenience and predictable pharmacokinetics. The RE-LY trial, conducted in 2009, demonstrated that dabigatran was non-inferior to warfarin for preventing stroke and systemic embolism, with a better safety profile, particularly in terms of major bleeding [34]. Despite these benefits, however, DOACs may not be suitable for all patients. For instance, dabigatran, while safe for those with mild-to-moderate renal impairment, requires careful dosing and is contraindicated in patients with severe renal impairment [35]. This is particularly relevant for elderly patients with renal dysfunction, who may be more vulnerable to adverse drug reactions. This highlights critical consideration in selecting the appropriate anticoagulant based on patient history.

As mentioned above, traditional anticoagulants such as warfarin and unfractionated heparin require regular monitoring of anticoagulant activity (e.g., INR for warfarin, aPTT for heparin) and dose adjustments to maintain therapeutic efficacy while minimizing bleeding risk. This monitoring can be time-consuming, cost ineffective, and may pose logistical challenges for patients.

DOACs generally do not require routine monitoring but may necessitate dose adjustments based on clinical factors such as renal function or body weight. However, despite their convenience, adherence to DOACs can still pose a challenge. For instance, patients on DOACs may find it difficult to keep track of their daily dosages, especially if they are experiencing multiple comorbidities that require additional medications. In fact, a study examining adherence to DOACs in France found that provider follow-up and experienced side effects of the medication, although minimal, were key adversaries to optimal therapy conditions, underscoring the importance of continuing care and patient education [36]. Another major obstacle to the use of DOACs is their cost, with some patients unable to afford these medications due to a lack of insurance coverage or high out-of-pocket expenses [37]. This has led to some patients abandoning therapy or switching to older, cheaper options like warfarin, which may not be ideal in the long term.

The risk of thrombosis and bleeding events is dynamic and can change over time due to factors such as acute illness, surgery, trauma, or changes in concomitant medications. Clinicians must continuously reassess and adjust anticoagulant therapy to mitigate these risks.

Patient preferences, values, and adherence to prescribed anticoagulant therapy play a crucial role in achieving treatment success. Patients may have concerns about the risk of bleeding, side effects of medication, dosing regimen, or the inconvenience of monitoring. Effective communication and shared decision-making between healthcare providers and patients are essential for addressing these concerns and optimizing treatment adherence. For instance, explaining the availability of reversal agents for DOACs (such as idarucizumab or andexanet alfa) may alleviate some patient concerns about bleeding complications and help improve adherence. Open discussions about the trade-offs between convenience, safety, and cost can help ensure that patients make informed choices tailored to their preferences and health needs.

Overall, achieving a balance between thrombosis prevention and minimizing bleeding risk requires a multifaceted approach that considers individual patient characteristics, treatment options, monitoring strategies, and ongoing risk assessment and management. The challenges in adherence, patient education, and the evolving landscape of anticoagulation management highlight the need for continuous monitoring and personalized care strategies. Collaboration between healthcare providers, patients, and other stakeholders is essential to navigate these challenges and optimize clinical outcomes in anticoagulant therapy.

## 3. Emerging Trends in Anticoagulation Management

### 3.1. New Oral Anticoagulants in Development

There are a few pharmacological candidates currently in development. Activated factor XI (FXIa) is a serine protease involved in the amplification of thrombin production [38]. It is a part of the intrinsic pathway of the coagulation cascade and is believed to play a significant role in thrombosis with a lesser extent in hemostasis, unlike thrombin and FXa. While elevated FXI levels have been linked to a higher risk of thrombosis, FXI deficiency may correspond to a reduced risk of cardiovascular events and venous thromboembolism [39]. The appeal of FXIa as a therapeutic target for anticoagulation is bolstered by the minimal bleeding associated with FXIa modulation. Additionally, hemorrhage following trauma or surgical procedures occurs less frequently and to a lesser extent in FXI-deficient patients compared to those with other types of coagulation factor deficiencies [40].

These new factor XIa (FXIa) inhibitors represent a promising area of development in anticoagulant therapy, aiming to provide more targeted and effective options for preventing thrombosis while potentially reducing bleeding risks compared to traditional anticoagulants. As mentioned earlier, FXIa plays a crucial role in the intrinsic coagulation pathway, which contributes to thrombus formation. By selectively inhibiting FXIa, these inhibitors intervene at an earlier stage of the coagulation cascade, potentially offering advantages in preventing thrombotic events [41]. Several FXIa inhibitors are currently in various stages of development, with ongoing clinical trials evaluating their safety and efficacy profiles. These novel agents hold the potential for addressing unmet needs in thrombosis management, particularly in patients at high risk of thrombotic events where traditional anticoagulants may not provide adequate protection. However, further research is needed to fully elucidate their therapeutic benefits and safety profiles before they can be widely adopted in clinical practice. Table 2 summarizes the development of FXIa inhibitors.

### 3.2. Personalized Anticoagulation Management

Personalized anticoagulation management represents a significant advancement in optimizing therapeutic outcomes and minimizing adverse effects in patients requiring anticoagulation therapy. One promising approach is pharmacogenomic testing, which tailors anticoagulant therapy based on an individual’s genetic makeup. Variations in genes such as CYP2C9, VKORC1, and CYP4F2 significantly influence the metabolism and efficacy of warfarin, a commonly used anticoagulant. By identifying these genetic variants through pharmacogenomic testing, clinicians can predict the optimal warfarin dose for each patient, thereby reducing the risk of over-anticoagulation or under-anticoagulation associated with incorrect dosing. Several studies have demonstrated that pharmacogenomic-guided warfarin dosing improves therapeutic outcomes and reduces the time to reach stable anticoagulation compared to standard dosing methods [51,52]. Pharmacogenomics of anticoagulants is further discussed below in conjunction with artificial intelligence.

### 3.3. Point-of-Care Devices

In addition to pharmacogenomic testing, the development and implementation of point-of-care (POC) offers rapid and convenient monitoring of anticoagulation status. These devices allow for real-time measurement of coagulation parameters such as international normalized ratio (INR), prothrombin time (PT), and activated partial thromboplastin time (aPTT) at the patient’s bedside or in outpatient settings. The immediacy and ease of use provided by POC devices enhance patient compliance and facilitate timely adjustments in anticoagulant dosing, thereby improving overall management. Furthermore, coupling POC devices with digital health platforms would enable continuous remote monitoring and real-time data sharing with healthcare providers, ensuring better patient oversight and intervention when necessary. Recent advancements in POC technology have led to the development of portable and user-friendly devices with high accuracy and reliability, demonstrating their potential to transform anticoagulation management. There are numerous papers discussing point-of-care coagulation testing in great detail [53,54,55,56].

## 4. Future Direction of Anticoagulation Management

### 4.1. Development of Anticoagulants with Enhanced Efficacy and Safety Profile

Future directions in anticoagulant development are to address the limitations of current therapies, such as the risk of bleeding complications and the need for frequent monitoring. Researchers are exploring novel targets within the coagulation cascade, like Factor XII or platelet-targeted therapies, to develop anticoagulants and antiplatelets with greater efficacy and safety. Additionally, advancements in drug delivery systems, such as targeted nanoparticles or gene therapy-based approaches, hold promise for improving the pharmacokinetic profiles and reducing the side effects of future anticoagulants. For instance, studies have investigated RNA aptamers targeting Factor XI for their anticoagulant properties, potentially offering safer alternatives to traditional anticoagulants by selectively inhibiting thrombosis without affecting hemostasis [57,58,59,60].

### 4.2. Artificial Intelligence and Pharmacogenomics in Anticoagulation

Artificial intelligence (AI) holds significant potential to revolutionize anticoagulation management by leveraging data analytics, predictive modeling, and decision support tools. Potential AI application examples in this field are:

- **Risk Prediction and Personalized Therapy**: AI algorithms can analyze patient data (clinical history, laboratory results, genetic information, imaging studies, etc.) to predict individualized thrombotic and bleeding risks. This personalized approach would help clinicians guide the selection of the most appropriate anticoagulation therapy and dosing regimens tailored to each patient’s risk profile.
- **Dose Optimization and Monitoring**: AI-powered algorithms can optimize anticoagulant dosing based on real-time patient data, such as drug levels, coagulation parameters, and clinical outcomes. By analyzing ongoing responses, AI systems can adjust dosages to achieve optimal anticoagulation while minimizing side effects. Machine learning models, such as artificial neural networks and reinforcement learning techniques, have been used to develop predictive dosing tools that adjust anticoagulant regimens dynamically, improving efficacy and reducing adverse effects [61].
- **Early Detection of Thrombotic and Bleeding Events**: AI-based models have the potential to detect subtle changes in patient data indicative of thrombotic or bleeding events before they manifest clinically. By dynamically analyzing coagulation, AI systems can alert healthcare providers to potential complications, enabling early intervention and prevention strategies. For instance, machine learning models have been employed to predict suboptimal anticoagulation control in patients with atrial fibrillation by analyzing clinical data, including patient demographics, comorbidities, and prior anticoagulation responses. Techniques such as Long Short-Term Memory (LSTM) recurrent neural networks and XGBoost have demonstrated the ability to identify patients at risk of inadequate anticoagulation, thereby enabling earlier intervention strategies to optimize therapy and reduce the likelihood of thrombotic or bleeding complications [62].
- **Treatment Adherence**: AI-powered applications can improve patient adherence to anticoagulation therapy by providing personalized education, reminders, and support tools. This capability of AI is already being actively incorporated into healthcare systems around the globe. For example, a machine learning-based mobile application has been tested on Indian patients with vitamin K antagonists, allowing them to manage their anticoagulation therapy without frequent physician visits. This smartphone-based system, which predicts warfarin doses based on PT-INR values, has demonstrated a high correlation with physician-prescribed doses and may enhance accessibility, particularly in low-resource settings. By reducing the logistical barriers to regular monitoring, AI-driven mobile health (mHealth) tools are helping improve adherence rates among patients on warfarin and other anticoagulants [63]. These applications can also facilitate remote monitoring and communication between patients and healthcare providers, enhancing patient engagement and therapeutic satisfaction.
- **Clinical Decision Support Systems**: AI-driven decision support systems can assist healthcare providers in making informed decisions by analyzing vast amounts of data and providing real-time recommendations on therapy choices, dosing adjustments, and monitoring strategies. For instance, a deep learning-based CDSS has been created to predict PT/INR values and generate individualized warfarin dosing recommendations, outperforming expert physicians in accuracy. This AI-powered platform integrates patient-specific data and simulates the effects of different dosing regimens, allowing clinicians to make more informed adjustments. When incorporated into hospital-based electronic prescribing systems, such CDSS tools have the potential to reduce errors in warfarin prescription, minimize adverse drug events, and improve the overall safety of anticoagulation therapy [64].
- **Quality Improvement and Global Health Management**: AI analytics can analyze large datasets to identify trends and opportunities for improvement in anticoagulation management across multiple healthcare systems. By analyzing outcomes, resource utilization, and adherence patterns across healthcare systems, AI systems can inform strategies to optimize care delivery and improve patient outcomes on a broader, perhaps global scale.
- **Pharmacogenomic-Guided Dosing Algorithms**: As mentioned above, patients with certain polymorphisms of genes responsible for anticoagulant metabolism require lower doses to prevent excessive anticoagulation and bleeding risks [51]. AI-driven models integrating genetic, clinical, and demographic data can be used to optimize anticoagulant dosing. For instance, the EU-PACT trial showed improved time in the therapeutic range with genotype-guided dosing [65]. The pharmacogenomic approach is true for the novel DOACs as well [66]. A study by Ji et al. demonstrated polymorphisms in ABCB1 and CES1 genes are linked to variations in dabigatran absorption and metabolism [67]. AI-driven pharmacogenomic tools may help further refine DOAC selection and dosing, especially in populations with known genetic risk factors [68]. These findings underscore the need for AI-driven dosing to enhance anticoagulation safety and reduce variability.

The combination of AI and pharmacogenomics holds promise for real-time, precision-guided anticoagulation therapy. AI models trained on large-scale pharmacogenomic datasets could predict individual responses to anticoagulants, reducing the trial-and-error approach in anticoagulation management. As AI-powered pharmacogenomics continues to evolve, it may pave the way for fully personalized anticoagulation regimens tailored to a patient’s genetic makeup, clinical profile, and lifestyle factors [65].

## 5. Challenges in Future Anticoagulation Management

### 5.1. Regulatory Hurdles for New Anticoagulants and Monitoring Technologies

Our understanding of basic science and coagulation processes has expanded in recent decades, setting higher therapeutic standards by developing more specific drugs that are potentially more effective and safer. The Food and Drug Administration (FDA) follows a strict policy when it comes to approving new medications. In past decades, the US market has experienced delays in the use of medical products due to multiple regulatory standards compared to Europe. This potentially may slow down the introduction of new anticoagulants to the local market.

The approval process begins with pre-clinical experiments conducted on cellular and animal models. This is followed by tests on human subjects (phases I, II, and III of clinical trials), lasting 6 to 10 years. At this point, institutions spend an average of 48 million US dollars per drug, and only one-fifth of these projects are approved by the FDA [69]. In addition, a clinical trial may be canceled at any time if significant harmful effects are discovered/reported.

Once the previous steps are completed, a more comprehensive understanding of safety and tolerance is obtained through post-market surveillance (phase IV). At this stage, some potentially harmful drugs may require pharmaceutical companies to implement risk evaluation and mitigation strategy programs that are cornerstones for patient safety. This includes but is not limited to patient education or interventions to prevent adverse effects. The “story” of dabigatran could serve as a good example. In 2010, after the introduction of dabigatran to the US market, severe bleeding events were observed in patients. This prompted the FDA to require additional risk evaluations and, ultimately, the development of an antidote (idarucizumab) for emergency reversal of its anticoagulant effects [70]. Consequently, manufacturers faced lawsuits based on incomplete disclosure of risks related to the drug. Such pitfalls with labeling and advertising shall be considered by manufacturers in the future while releasing new blood thinners to acknowledge all possible risks to customers.

As we know, generic medications typically increase accessibility due to cost reduction. However, the FDA sets requirements that demand all generics demonstrate bioequivalence and the same safety profiles. One caveat is that patent extensions can delay the availability of generic medications. It is also worth mentioning that new anticoagulants may require monitoring, which could require novel methodologies and, therefore, will need calibration using specialized laboratory testing. Such tools must be validated in the clinical setting according to FDA regulations. Additionally, clinicians and medical societies will need to reach a consensus regarding new therapeutic parameters. Therefore, manufacturers of anticoagulant medications need to consider these aspects when developing future anticoagulation medications.

### 5.2. Cost-Effectiveness Considerations in Novel Therapies

Cost-effectiveness in healthcare refers to evaluating the relative costs and effects (clinical outcomes) of different courses of action. In healthcare, it is important to ensure that resources are used efficiently to achieve the best possible health outcomes for a given society. Whether it is a developing country battling infectious diseases or a well-developed nation facing an aging population, health economics provides a basic framework for maximizing the health outcomes of a given social entity within its budgetary constraints. This concept helps policymakers and healthcare providers make informed decisions about which treatments to fund, ensuring that the benefits of medical interventions would justify their costs.

In anticoagulation management, cost-effectiveness is particularly important due to the high prevalence of conditions like atrial fibrillation, especially in countries with aging populations. Given the lifelong requirement for anticoagulation in many of these patients, assessing the cost-effectiveness of the therapies helps determine the most economically sustainable and clinically effective options for preventing strokes and other cardiovascular events.

Traditional anticoagulants like warfarin have been the mainstay of therapy for decades. They are effective but require regular monitoring and have numerous dietary and drug interactions. DOACs offer several advantages, such as fewer interactions and no need for routine monitoring. The primary benefits of DOACs include reduced rates of stroke and systemic embolism, as well as potentially lower rates of major bleeding compared to warfarin. However, these benefits must be weighed against significantly higher drug costs. Studies have shown that while DOACs may be more expensive initially, their use can be justified if they lead to better health outcomes, lower overall healthcare costs due to no need for regular monitoring, and fewer complications and hospitalizations [71].

Economic evaluations in healthcare include cost-effectiveness analysis (CEA) and cost-benefit analysis (CBA). CEA compares the cost of anticoagulation medications to their health outcomes. In a recent study, it was found that all of the DOACs but rivaroxaban were superior to warfarin in CEA for the prevention of stroke in patients with atrial fibrillation [72]. Conversely, CBA compares the total costs (both healthcare and societal) of using DOACs to the total benefits (increased productivity, reduced societal burden of illness). Another recent study demonstrated that DOACs tested were more effective and more cost-effective than LMWH in the prevention of cancer-associated thrombosis [73].

The cost-effectiveness of anticoagulant therapies varies based on drug prices, patient characteristics, clinical settings, and local healthcare practices. Future research should focus on long-term data and real-world outcomes specific to clinical settings to better understand the cost-effectiveness of DOACs, including the novel FXI/FXIa inhibitors that are about to enter the market.

Continuous monitoring of drug prices and evolving clinical evidence will be essential for updating healthcare policies and guidelines to ensure the optimal use of resources in anticoagulation therapy. Personalized treatment approaches and further cost-effectiveness analyses will help tailor therapies to individual patient needs, enhancing the overall quality and efficiency of healthcare delivery.

### 5.3. Data Privacy and Security Concerns with Digital Anticoagulation

As anticoagulation management becomes increasingly digitized, ensuring data privacy and security is a critical step in the process. Digital systems collect and analyze sensitive patient information in order to offer personalized, individual care and real-time monitoring, making robust data protection measures essential. There are a few points to consider:

Data Breaches: The healthcare sector is a prime target for data breaches due to the sensitive nature of the data that it possesses [74]. These types of cyberattacks can result in unauthorized access and collection of patient information, leading to privacy violations and potential misuse of personal data. The consequences of such breaches are severe, impacting patient trust and potentially leading to severe legal and financial ramifications for healthcare facilities and providers. As a result, patients must trust that their data will be used ethically and protected from unauthorized access.

Data Integrity: Maintaining accurate and consistent patient data is crucial for effective anticoagulation management [75]. Digital systems must ensure that the data are not altered or tampered with, as any discrepancies can lead to incorrect treatment decisions (such as inaccurate dosage adjustments) and, thus, negative patient outcomes. Ensuring data integrity involves implementing strict verification processes and regular audits to detect and minimize any errors or discrepancies efficiently. Errors also appear in the form of large datasets, which pose significant analytical challenges and must be interpreted correctly to avoid false conclusions leading to inappropriate healthcare measures.

Security Measures: To safeguard patient data, healthcare organizations must implement robust security measures such as encryption, multi-factor authentication (MFA), and secure data transmission protocols. These measures help protect data from unauthorized access and cyber threats. Additionally, ongoing security training for healthcare staff is essential in mitigating risks related to human error and insider threats, safely and properly handling these sensitive data, and adapting to evolving threats and vulnerabilities.

Compliance with Regulatory Bodies: Healthcare organizations need to be able to navigate the complex regulatory landscape to ensure compliance with data security and privacy laws on a larger scale. Regulatory bodies such as the Health Insurance Portability and Accountability Act (HIPAA) in the United States [76], the General Data Protection Regulation (GDPR) in the European Union [77], and other regional laws mandate rigorous data protection standards. Compliance involves but is not limited to continuous monitoring, risk assessments, and adherence to best data management and security practices. These standards, mandates, and recommendations must be considered while digitalizing anticoagulation management.

Advantages: Although digital anticoagulation management presents significant privacy and security challenges, there are notable benefits to opening up health data for research purposes [78]. Such transparency can enrich data on symptoms, diseases, diagnoses, and treatment, offering potential novel improvements in individual and population care. It also facilitates the discovery of unknown symptoms and personalized treatments, leading to a deeper understanding of the multifaceted nature of health outcomes and healthcare delivery challenges. Additionally, easier access to information enables opportunities for home care using remote and telehealth technologies, especially concerning long-term conditions.

Disadvantages: However, ownership of personal data raises critical issues. While opening data for research drives new opportunities, it also demands a balance between individual privacy and the need for evidence to drive healthcare improvements. Citizens are becoming increasingly concerned about the direct collection of their private health data, often without the opportunity to opt out of this feature. These data, therefore, can be subject to intrusion through unregulated sharing and use.

Digitalization enhances anticoagulation monitoring, provides personalized treatment plans, and improves patient outcomes by providing a more data-driven, individualized approach. However, addressing data privacy and security challenges is essential to fully realize these benefits and build patient trust with this addition to the healthcare system [79]. Implementing comprehensive security measures, maintaining data integrity, and ensuring regulatory compliance are critical steps in safeguarding patient information and maintaining trust in digital healthcare systems.

## 6. Conclusions

In summary, anticoagulation management encompasses a diverse array of pharmacological approaches, each with distinct mechanisms of action and therapeutic roles in preventing thrombosis. From the pioneering days of vitamin K antagonists to the modern era of direct oral anticoagulants (DOACs) and emerging therapies like FXIa inhibitors, continuous innovation is shaping the landscape of anticoagulation management. Challenges such as individual variability in response, the need for vigilant monitoring, and balancing thrombotic prevention with bleeding risks highlight the complexity of clinical decision-making in anticoagulation therapy.

Looking into the future, advancements in the personalized medicine niche of anticoagulation management, artificial intelligence, and novel anticoagulant development hold promise for optimizing therapeutic outcomes while addressing current limitations. However, alongside these promising developments, ensuring robust data privacy and security measures in increasingly digitalized healthcare environments remains paramount to fostering patient trust and realizing the full potential of digital healthcare systems in anticoagulation management.

## Figures and Tables

**Table 1:** FDA-Approved Anticoagulants.

Anticoagulant	Mechanism of Action	Administration Route	Common Side Effects	Half-Life (h)	Monitoring
Warfarin	Inhibits vitamin K epoxide reductase, thereby reducing the synthesis of vitamin K-dependent clotting factors (II, VII, IX, X)	Oral	Bleeding, skin necrosis, hair loss, drug interactions	20–60	PT/INR monitoring, typically every 2–4 weeks initially, then less frequent once stable
Other Vitamin K Antagonists (e.g., Acenocoumarol, Phenprocoumon)	Inhibits vitamin K epoxide reductase, reducing the synthesis of vitamin K-dependent clotting factors	Oral	Bleeding, skin necrosis, drug interactions	20–60	
Heparin	Binds to antithrombin III, enhancing its ability to inactivate thrombin (factor IIa) and Xa, and to a lesser extent VIIa, IXa, XIa	Intravenous	Bleeding, heparin-induced thrombocytopenia (HIT), osteoporosis	1–2	aPTT (Activated Partial Thromboplastin Time) monitoring for unfractionated heparin; Anti-Xa assay for LMWH
Low Molecular Weight Heparin (LMWH)	Similar to unfractionated heparin but with greater inhibition of factor Xa than thrombin	Subcutaneous	Bleeding, HIT, injection site reactions	4–5	Anti-Xa assay
Fondaparinux	Selectively inhibits factor Xa indirectly through binding to antithrombin III. No direct inhibitory effect on thrombin	Subcutaneous	Bleeding, HIT, injection site reactions	17–21	
Danaparoid	Selectively inhibits factor Xa and factor IIa (thrombin) indirectly through binding to antithrombin III	Intravenous	Bleeding, HIT	23–26	
Direct Thrombin Inhibitors (e.g., Dabigatran)	Selectively inhibits thrombin (factor IIa), preventing fibrin formation	Oral	Bleeding, gastrointestinal discomfort, dyspepsia	12–17	No routine monitoring required
Factor Xa Inhibitors (e.g., Rivaroxaban, Apixaban, Edoxaban, Betrixaban)	Selectively inhibits factor Xa, thereby preventing the conversion of prothrombin to thrombin	Oral	Bleeding, gastrointestinal symptoms, hepatotoxicity	5–27	
Intravenous Direct Thrombin Inhibitors (Argatroban, Bivalirudin)	Selectively inhibits thrombin (factor IIa), preventing fibrin formation	Intravenous	Bleeding, thrombocytopenia (bivalirudin), allergic reactions, liver dysfunction (argatroban)	0.5–1	aPTT, ecarin clotting time (ECT), activated clotting time (ACT)

**Table 2:** FXI-Anticoagulants in Development.

Anticoagulant	Mechanism of Action	Administration Route	Developing Company	Development Stage
Fesomersen	Factor XI antisense oligonucleotide [42]	Subcutaneous	Ionis Pharmaceuticals	Clinical Trials Phase II
Milvexian	Small-molecule factor XIa inhibitor [43]	Oral	Bristol Myers Squibb	Clinical Trials Phase III
Asundexian	Small-molecule factor XIa inhibitor [44]	Oral	Bayer	Clinical Trials Phase III
BMS-962212	Direct, reversible, selective factor XIa inhibitor [45]	Intravenous	Bristol Myers Squibb	Undisclosed
ONO-7684	Small-molecule factor XIa inhibitor [46]	Intravenous	ONO Pharmaceuticals	Undisclosed
Abelacimab	A monoclonal antibody that binds to factor XI and locks it in zymogen form [47]	Oral	Anthos Therapeutics	Clinical Trials
Osocimab	A monoclonal antibody that binds adjacent to the active site of factor XIa and prevents it from activating factor IX [48]	Subcutaneous	Aronora, Bayer	Clinical Trials Phase II
Gruticibart (formerly AB023)	A monoclonal antibody that blocks contact activation of coagulation by inhibiting factor XI activation by factor XIIa but not by thrombin [49]	Subcutaneous	Aronora	Clinical Trials Phase II
SHR2285	Small-molecule factor XIa inhibitor [50]	Intravenous	Jiangsu HengRui	Clinical Trials Phase II
REGN7508	A monoclonal antibody against the catalytic domain of factor XI	Intravenous, Subcutaneous	Regeneron	Clinical Trials Phase II
REGN9933	A monoclonal antibody against the A2 domain of factor XI			Clinical Trials Phase II

## Abbreviations

The following abbreviations are used in this manuscript:

aPTT	activated partial thromboplastin time
ACT	activated clotting time
CABG	coronary arterial bypass grafting
CBA	cost-benefit analysis
CEA	cost-effectiveness analysis
CYP2C9	cytochrome P450 family 2 subfamily C member 9
CYP4F2	cytochrome P450 family 4 subfamily F member 2
DOAC	direct oral anticoagulant
DTI	direct thrombin inhibitor
DVT	deep venous thrombosis
ECMO	extracorporeal membrane oxygenation
ECT	ecarin clotting time
FDA	Food and Drug Administration
FXa	activated factor Xa
FXIa	activated factor XI
GLA	Vitamin K-dependent carboxylation/gamma-carboxy glutamic domain
INR	international normalized ratio
IV	intravenously
LMWH	low molecular weight heparin
PCI	percutaneous coronary intervention
PE	pulmonary embolism
PIVKA	protein induced by vitamin K absence
PT	prothrombin time
SC	subcutaneously
QALY	quality-adjusted life years
UFH	unfractionated heparin
VKA	vitamin K antagonist
VKORC1	vitamin K epoxide reductase complex 1
VTE	venous thromboembolism
